# Virus-triggered spinal cord demyelination is followed by a peripheral neuropathy resembling features of Guillain-Barré Syndrome

**DOI:** 10.1038/s41598-019-40964-1

**Published:** 2019-03-14

**Authors:** Eva Leitzen, Barbara B. Raddatz, Wen Jin, Sandra Goebbels, Klaus-Armin Nave, Wolfgang Baumgärtner, Florian Hansmann

**Affiliations:** 1 Department of Pathology, University of Veterinary Medicine Hannover, Bünteweg 17, 30559 Hannover, Germany; 2 Center for Systems Neuroscience, Hannover, Germany; 3 Department of Neurogenetics, Max-Planck-Institute for Experimental Medicine, Hermann-Rein-Straße 3, 37075 Göttingen, Germany

## Abstract

Theiler’s murine encephalomyelitis virus (TMEV)-induces a demyelinating disease in the spinal cord (SC) of susceptible but not in resistant (B6) mouse strains. The aim of the present study was to induce SC demyelination and a peripheral neuropathy in resistant mice by switching the infection site from cerebrum to SC. B6 mice were intraspinally inoculated with TMEV. Infected mice showed clinical signs starting at 7 days post infection (dpi). Histopathology revealed a mononuclear myelitis, centred on the injection site at 3 dpi with subsequent antero- and retrograde spread, accompanied by demyelination and axonal damage within the SC. Virus protein was detected in the SC at all time points. SC inflammation decreased until the end of the investigation period (28 dpi). Concurrent with the amelioration of SC inflammation, the emergence of a peripheral neuropathy, characterized by axonal damage, demyelination and macrophage infiltration, contributing to persistent clinical sings, was observed. Intraspinal TMEV infection of resistant mice induced inflammation, demyelination and delayed viral clearance in the spinal cord and more interestingly, subsequent, virus-triggered inflammation and degeneration within the PN associated with dramatic and progressive clinical signs. The lesions observed in the PN resemble important features of Guillain-Barré syndrome, especially of acute motor/motor-sensory axonal forms.

## Introduction

Theiler’s murine encephalomyelitis virus (TMEV) was firstly described as a neuropathogenic virus and causative agent of Theiler’s murine encephalitis (TME) in the 1930s by Max Theiler^1,2^. After experimental intracerebral (i.c.) infection of susceptible mouse strains (e.g. SJL) with the low virulent BeAn-strain of TMEV, animals develop a biphasic disease course consisting of an acute polioencephalitis (early disease) followed by virus persistence associated with chronic demyelination within the spinal cord white matter starting between two and six weeks after infection^3–6^. Resistant mouse strains like C57BL/6 (B6) are capable of clearing TMEV from the central nervous system (CNS) during the acute phase of the disease. Thereby, the development of virus persistence, subsequent clinical signs and chronic demyelination within the SC is prevented^7,8^. The development and progression of TME is determined by numerous factors including the genetic background and the immune response^9–11^. Genetic factors contributing to resistance of B6 mice to the development of TMEV-induced demyelinating disease are well known including a more efficient antiviral immune response^12–15^. In this context, numerous studies describe a compartmentalization of the immune response between CNS and periphery during infectious as well as inflammatory diseases^16–18^. Furthermore, significant differences regarding the immune response and glial cell reaction within brain and spinal cord exist which are attributed to various factors including differences between blood-brain barrier (BBB) and blood-spinal cord barrier (BSCB) as well as different reaction patterns of microglia/macrophages^19–22^. Following traumatic injury, B6 mice showed a more pronounced BSCB breakdown as well as higher numbers and a more widespread distribution of neutrophils and macrophages within the SC compared to the brain^20^. Therefore, a change of infection site in the present study from brain to spinal cord (SC) was considered as a possible adjusting parameter for a different disease outcome in resistant B6 mice, especially with regard to the extent of inflammation, demyelination and remyelination. During chronic TME in susceptible mice, remyelination is scarce^6,23^. The most popular theories explaining the ineffective regeneration postulate a block of oligodendrocyte precursor cell (OPC) differentiation and maturation^24,25^. OPCs can be identified by their expression of nerve/glial antigen 2 (NG2), also known as chondroitin sulfate proteoglycan 4 (CSPG-4). Migration to the lesion site as well as the maturation of OPCs plays an important role for the initiation and continuation of remyelination^26–28^. Moreover, TMEV injection into the sciatic nerve was followed by demyelination of the PN and virus spread to the SC with emergence of a demyelinating disease^29^. Several spontaneously occurring as well as experimentally induced animal models of acute inflammatory demyelinating and chronic inflammatory peripheral neuropathies have been described in the literature, even though most of them represent genetic or primarily autoimmune models and only few use viral infections including Gallid Herpesvirus and TMEV as trigger mechanism^29–32^. Some of the models show similarities to the Guillain-Barré syndrome (GBS), an entity with several subtypes characterised by demyelination of the PNS and subsequent neurological impairment^33,34^. The occurrence of GBS is often accompanied by an antecedent infection with bacterial (e.g. Campylobacter jejuni) or viral (e.g. cytomegalovirus, Zika virus) pathogens^33–36^. However, the primary antigenic target of demyelination in GBS is unknown and may be non-identical in affected individuals^30,31^.

The hypothesis of the present study is that direct intraspinal (i.s.) TMEV infection of resistant B6 mice will lead to demyelination in the SC as a result of a more pronounced inflammatory response compared to intracerebral infection. This would provide a reproducible mouse model to study virus-induced de- and remyelination at early time points mimicking lesions in susceptible mice during the late phase of TME. Moreover, there is the major advantage of harnessing a virus-induced demyelination model on B6 background, since most commercially available knock-in and knock-out mouse strains are only available on this genetic background. Therefore, the aims of this study were (1) to investigate TMEV-induced de- and remyelination in the SC of a resistant mouse strain, (2) to test the hypothesis of TMEV being capable of spreading to the PNS as well as (3) inducing a peripheral neuropathy after i.s. infection.

## Results

### Clinical investigation and motor coordination

**Figure 1:**
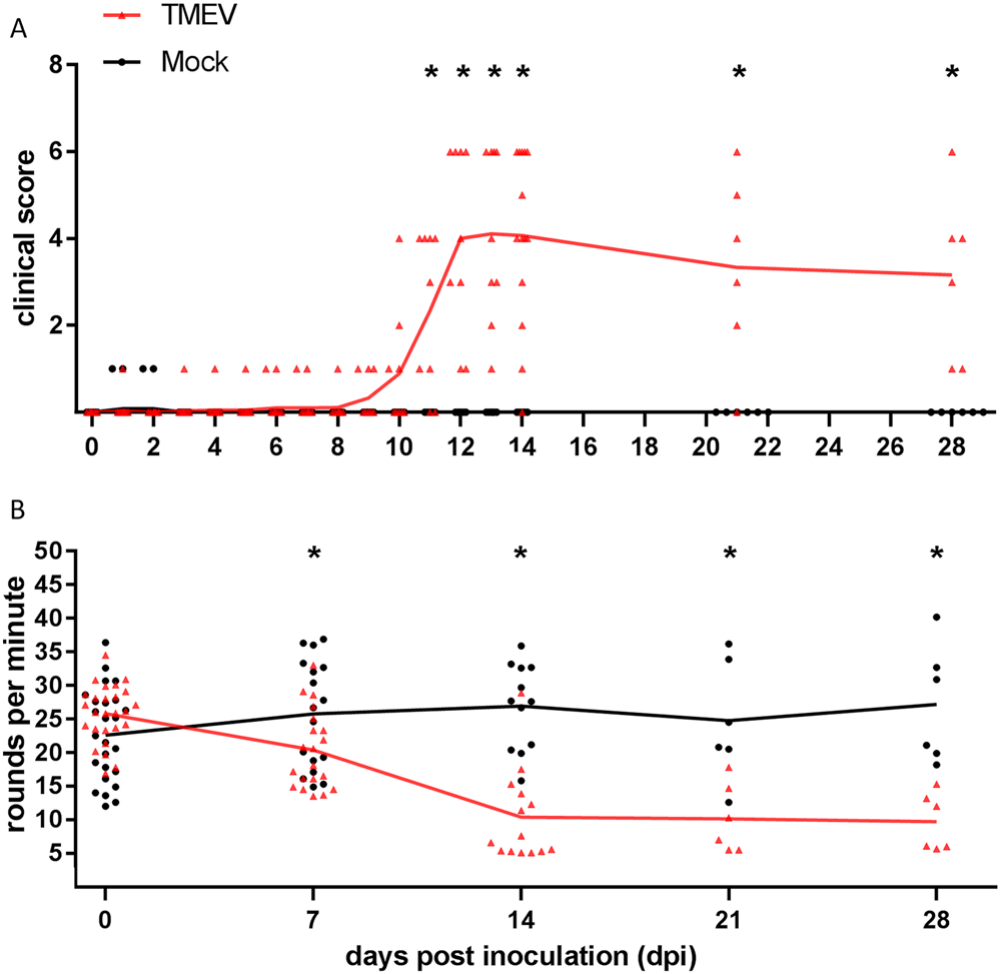
Clinical investigation of B6 mice following intraspinal TMEV infection. TMEV-infected mice showed significant clinical signs (**A**, starting at 11 dpi) and a deterioration of motor coordination and performance (**B**, starting at 7 dpi) compared to mock infected animals. The following number of animals was evaluated per group: n = 25–26 from 0 to 3 dpi; n = 17–20 from 4 to 7 dpi; n = 12–14 from 8 to 28 dpi. The graphs show mean (solid line) and individual value plots indicating the clinical score of each animal consisting of the sum of scores in the categories posture and external appearance, behaviour and activity as well as gait for clinical investigation or a mean round per minute value of three consecutive measurements for rotarod analysis, respectively. Significant differences between the groups as detected by Mann-Whitney U-test are marked by *asterisks* (p ≤ 0.05).

Clinical scores of TMEV-infected mice were significantly increased starting at 11 dpi until the end of the investigation period (Fig. 1A). Initial clinical signs were characterized by unilateral weakness and lameness, predominantly affecting the hind limbs. Rotarod analysis revealed a significant deterioration of motor coordination in TMEV-infected animals from 7 dpi until the end of investigation period (Fig. 1B).

### Spinal cord and peripheral organs

**Figure 2:**
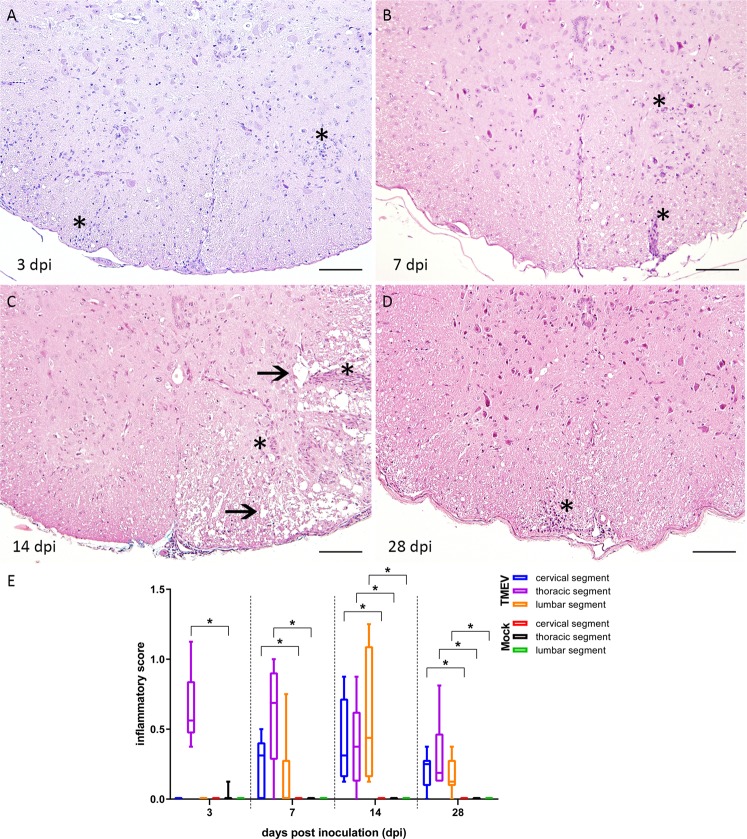
Histological changes within the thoracic spinal cord at 3 (**A**), 7 (**B**), 14 (**C**) and 28 (**D**) days post intraspinal infection with TMEV and the detection of an antero- and retrograde spread. (**E**) Lesions consisted of meningitis and perivascular accentuated lympho-histiocytic inflammation (**A**–**D**, asterisks) as well as multifocal dilated myelin sheaths and demyelination indicated by loss of eosinophilia in the white matter (**C**, arrows). The following number of animals was evaluated per group: n = 6–8 at 3 dpi; n = 5–6 at 7 dpi; n = 6–8 at 14 dpi; n = 6 at 28 dpi. Box-and-whisker plots show median and quartiles. Significant differences between the groups as detected by Mann-Whitney U-test are marked by asterisks (p ≤ 0.05). HE, bars = 100 µm.

Evaluation of HE stained SC slides revealed increasing numbers of inflammatory cells in TMEV-infected animals. Infected animals showed a polio- and leukomyelitis with predominance of the lesions within the ventral part of the white matter. At all investigated time points TMEV-infected animals showed a significant inflammatory infiltration near the injection site compared to Mock infected animals (Fig. 2A–D). Cervical (rostral) and lumbar (caudal) segments were significantly affected but most prominent at later time points in TMEV-infected animals (14 and 28 dpi), indicating a retro- and anterograde dissemination of inflammation (Fig. 2E).

**Figure 3:**
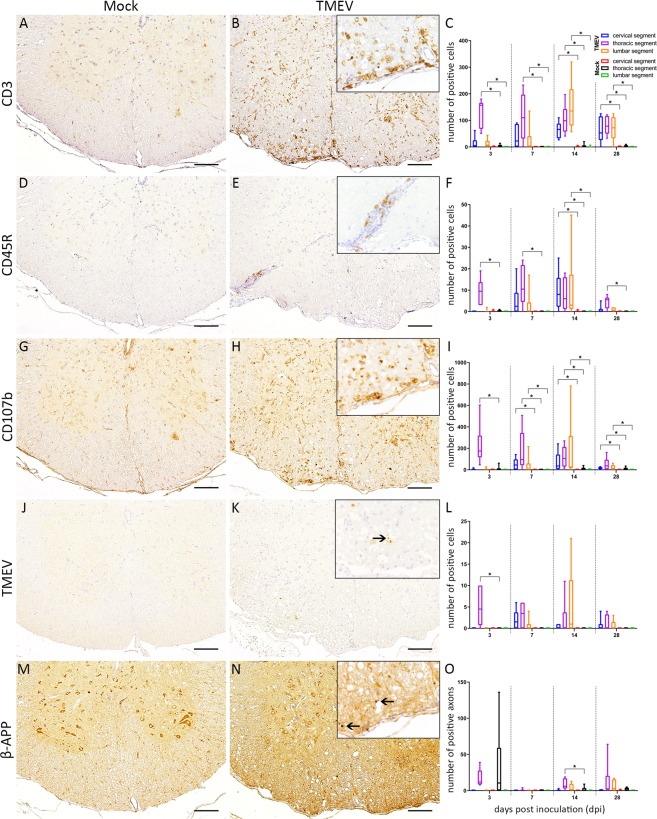
Immunophenotyping of inflammatory cells within the thoracic spinal cord at 14 days post intraspinal mock (**A**,**D**,**G**,**J**,**M**) and TMEV (**B**,**E**,**H**,**K**,**N**) injection. Statistical analysis revealed significantly increased numbers of CD3 (T lymphocytes), CD45R (B lymphocytes) and CD107b (microglia/macrophages) positive cells (**C**,**F**,**I**), the presence of virus protein (**K**, arrow) as well as an accumulation of beta-amyloid-precursor protein (β-APP; **N**, arrows) in the spinal cord of TMEV-infected animals. The following number of animals was evaluated per group: n = 6–8 at 3 dpi; n = 5–6 at 7 dpi; n = 6–8 at 14 dpi; n = 6 at 28 dpi. Box-and-whisker plots show median and quartiles. Significant differences between the groups as detected by Mann-Whitney U-test are indicated by *asterisks* (p ≤ 0.05), bars = 100 µm. Inserts in 400x magnification.

Immunohistochemistry was applied for immunophenotyping of inflammatory cells. At the injection site and in the lumbar segment a significant infiltration of T lymphocytes was observed at 3 and 7 dpi. At 14 and 28 dpi all investigated SC segments revealed a significantly increased number of T lymphocytes (Fig. 3A–C). The number of B lymphocytes was significantly increased around the injection site at all investigated time points as well as within cervical and lumbar segments at 14 dpi (Fig. 3D–F). The number of microglia/macrophages was significantly increased around the injection site at 3 dpi, in all investigated segments at 7 and 14 dpi and within cervical and lumbar segments at 28 dpi (Fig. 3G–I).

**Figure 4:**
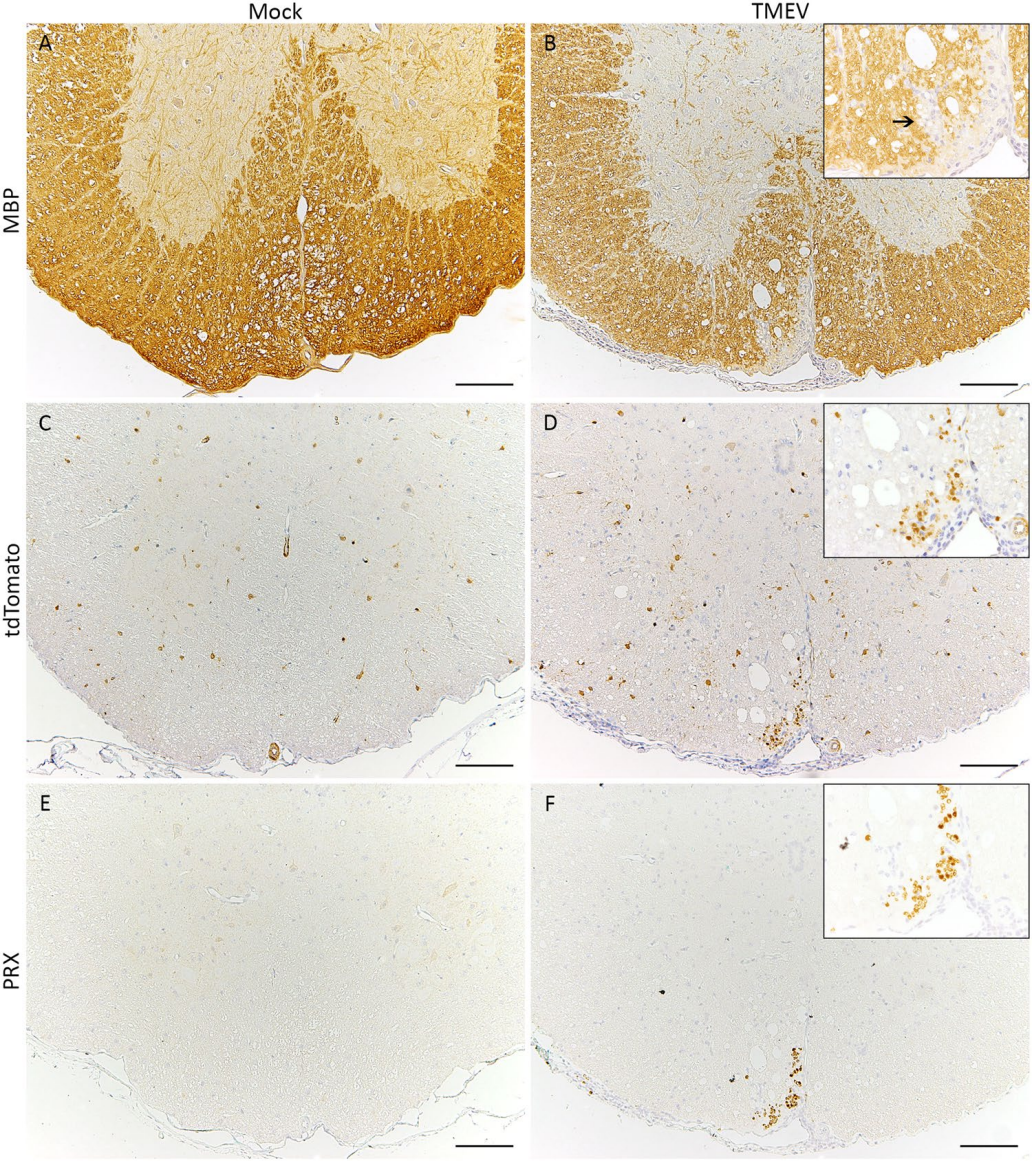
Detection of de- and potential approaches for remyelination. Demyelination (arrow) was identified using immunohistochemistry targeting myelin basic protein (MBP; **A**,**B**). Areas with inflammation and demyelination were infiltrated by tdTomato-positive glial cells as well as Periaxin-positive Schwann cells (arrowheads; **C**–**F**), bars = 100 µm. Inserts in 400x magnification.

Significant numbers of TMEV-positive cells were detected at the injection site at 3 dpi while virus spread to adjacent rostral and caudal segments was present at later time points (Fig. 3J–L). Accumulation of β-APP as a marker for axonal damage was significantly increased at 14 dpi around the injection site in TMEV-infected animals (Fig. 3M–O). Significant demyelination in the SC of TMEV infected animals was detected at 14 and 28 dpi (Fig. 4A,B). The quantification of tdTomato-positive cells at the injection site revealed increasing cell numbers in TMEV-infected and non-infected mice until the end of the investigation period. An accumulation of tdTomato-positive cells around demyelinated foci, predominantly located within the ventral aspect of the thoracic spinal cord, was observed at 14 and 28 dpi (Fig. 4C,D). Significantly increased numbers of td-Tomato-positive cells were detected within the thoracic segment of TMEV infected animals between 14 and 28 dpi. An accumulation of PRX-positive cells was detected around lesion sites in TMEV-infected animals while mock-infected mice did not show PRX-positive cells in the spinal cord (Fig. 4E,F). Decreasing inflammatory cell numbers within the SC at the end of the investigation period contrasted with the persistent deterioration of clinical signs in TMEV infected mice indicating additional causes for their clinical impairment. Histopathological investigation of peripheral organs (esophagus, trachea, thyroid, parathyroid, thymus, heart, spleen, liver, stomach, pancreas, small and large intestine, lymph nodes (mesenteric, subiliac, cervical, mandibular, lumbar, mediastinal), kidneys, genital organs including mammary gland, urinary bladder, skeletal muscle, skin, salivary gland, tongue, nose, bone and bone marrow) revealed no significant alterations in all animals. To further disclose the underlying mechanisms of clinical deterioration peripheral nerves were investigated in detail.

### Peripheral nerves

**Figure 5:**
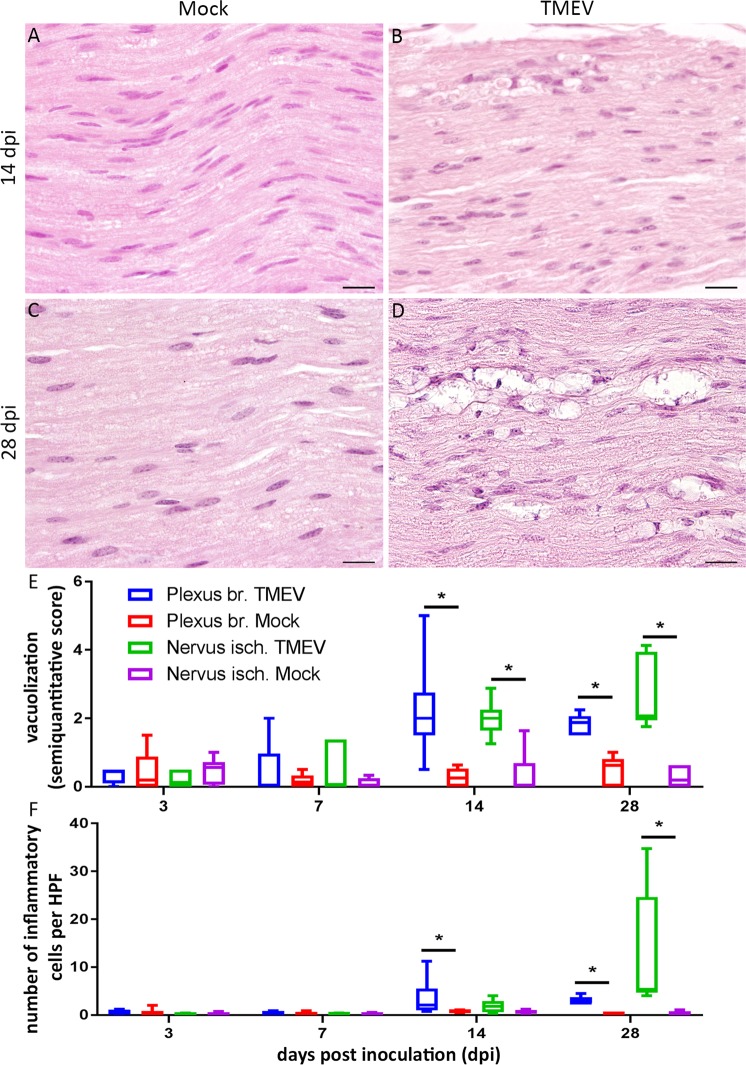
Investigation of peripheral nerves revealed a vacuolization of nerve fibers as well as an increased cellularity due to infiltration of inflammatory cells at 14 (**A**,**B**) and 28 (**C**,**D**) days post intraspinal TMEV infection. The number of inflammatory cells is presented as average number per high power field (HPF; **E**). For quantification of vacuolization, a semiquantitative scale was used (0 = no vacuoles; 1 =  ≤3 vacuoles per HPF); 2 =  <10% of nerve area per HPF affected, 3 = 10–20% of nerve area per HPF affected; 4 = 20–30% of nerve area per HPF affected; 5 = 30–40% of nerve area per HPF affected; (**F**). The following number of animals was evaluated per group: n = 6–8 at 3 dpi; n = 5–6 at 7 dpi; n = 6–8 at 14 dpi; n = 6 at 28 dpi. Data are presented as box-and-whisker plots showing median and quartiles. Significant differences between groups as detected by Mann-Whitney U-test were indicated by *asterisks* (p ≤ 0.05). Bars = 20 µm.

Degenerative and reactive changes including vacuolization, demyelination, spheroids, macrophages and myelinophagia were detected at the proximal and middle aspect of the PN of TMEV infected animals. Moreover, PN showed an increased cellularity at 14 and 28 dpi (Fig. 5).

**Figure 6:**
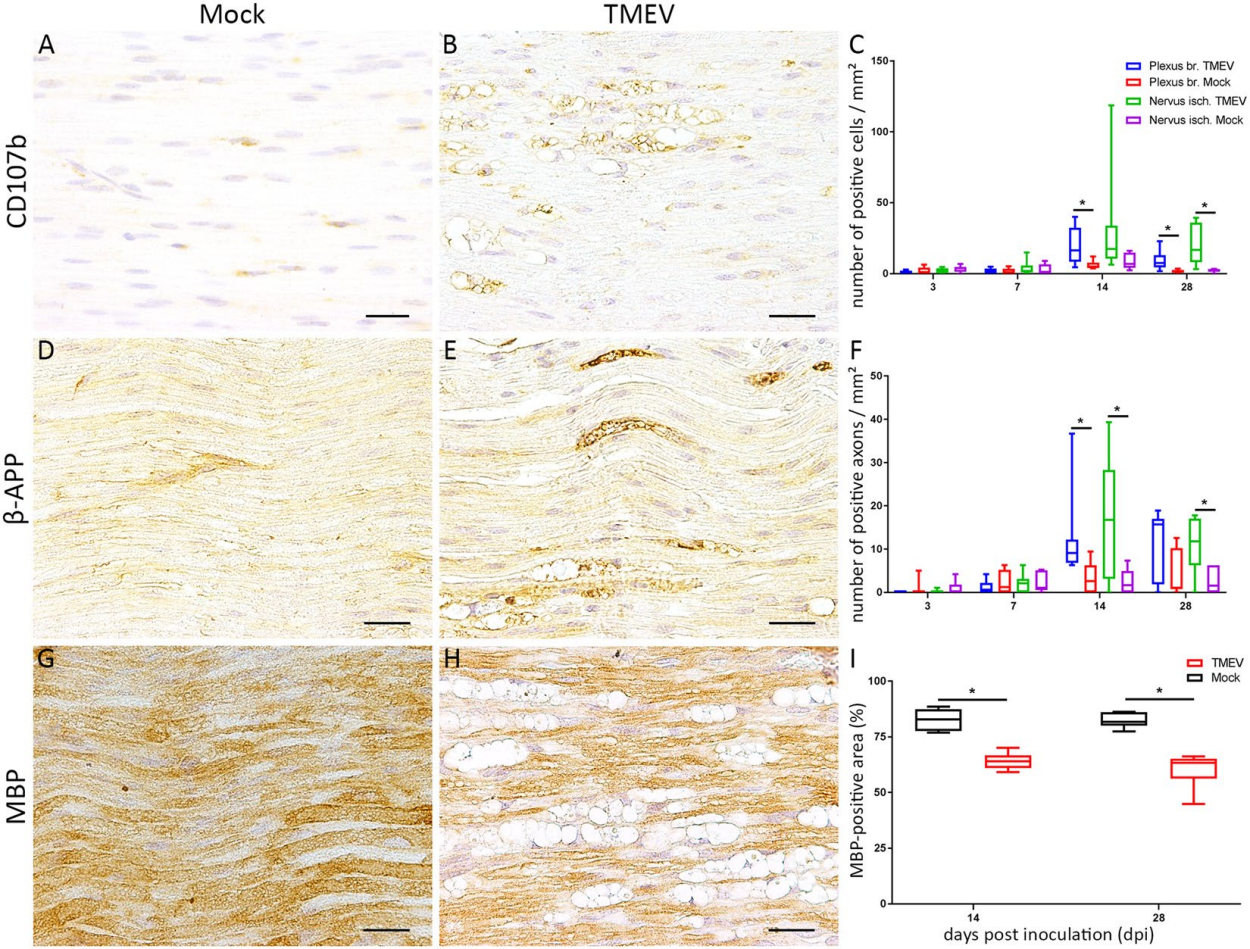
Quantification of macrophages (CD107b), axonal damage (beta-amyloid-precursor-protein, β-APP) and demyelination (myelin basic protein, MBP) in peripheral nerves at 28 days post inoculation (dpi). Inflammation consisting of an infiltration of macrophages within peripheral nerves was detected at 14 and 28 dpi (**A**–**C**). TMEV infected animals showed significant axonal damage (**D**–**F**) as well as demyelination (**G**–**I**) at 14 and 28 dpi. The following number of animals was evaluated per group: n = 6–8 at 3 dpi; n = 5–6 at 7 dpi; n = 6–8 at 14 dpi; n = 6 at 28 dpi. Data are presented as box-and-whisker plots showing median and quartiles. Significant differences between groups detected by Mann-Whitney U-test were indicated by *asterisks* (p ≤ 0.05). Bars = 20 µm.

Immunophenotyping of inflammatory cells in PN of TMEV-infected animals revealed an increased density of CD107b positive cells within the plexus brachiales at 14 dpi and within all PN at 28 dpi (Fig. 6A–C) while only single CD3 and/or CD45R positive cells were present (data not shown).

Significant accumulation of β-APP within PN of TMEV-infected animals was detected at 14 dpi in plexus brachiales and nervi ischiadici as well as within the nervi ischiadici at 28 dpi. (Fig. 5D–F). 2/26 TMEV infected animals showed single TMEV-positive fibers at 7 and 14 dpi, respectively. Significant demyelination as indicated by an increased MBP negative area was detected at 14 and 28 dpi.

## Discussion

Theiler’s murine encephalomyelitis induced demyelinating disease represents a well-established animal model for the progressive forms of multiple sclerosis and virus-mediated demyelination^8,37,38^. Since resistant mouse strains like B6 lack demyelinating CNS lesions following intracerebral infection the hypothesis of the present study was that an intraspinal infection would lead to demyelinating SC lesions similar to those observed in SJL mice during the chronic phase of TME^2,8,12,39,40^.

### Clinical investigation and motor coordination

Significant clinical impairment in intraspinally TMEV-infected B6 mice was firstly perceived as a decrease in rotarod performance at 7 dpi while a significant deterioration of clinical signs was observed at 11 dpi. This might indicate a higher sensitivity of the rotarod test compared to the applied clinical scoring system following intraspinal infection. In comparison, after i.c. infection of SJL-mice, animals showed marked reduction in motor strength as late as 42 to 70 dpi in association with virus persistence, SC inflammation, demyelination and axonal damage^23,41,42^. In contrast, intraspinally infected mice showed an earlier onset of clinical signs at 11 dpi, which included waddling gait, ataxia, spasticity and paralysis of hind limbs, matching the clinical features seen in SJL mice during the late phase of the disease^4,8,37^. The rather rapid deterioration of clinical signs between days 7 and 12 may be attributed to the combination of increasing SC inflammation and onset of PN lesions.

### Spinal cord

Composition and localization of inflammatory lesions consisted predominantly of T lymphocytes and microglia/macrophages, thereby mimicking the inflammatory pattern in susceptible mice during the late phase of TME^6,8,43^. The presence of B lymphocytes was less pronounced and mostly restricted to the injection site. Following intraspinal TMEV-infection inflammation as well as virus protein are mainly localized within the white matter. This is in contrast to studies investigating the acute phase of TME in B6 mice following i.c. infection^44^. B6 mice are known for being capable of clearing TMEV from the CNS within approximately 2 to 3 weeks post i.c. infection^8,45^. In the present study TMEV antigen was detectable - even though scarcely - in SC tissue at 28 dpi. This raises the question why the elimination of TMEV following intraspinal infection is delayed in comparison to virus elimination following intracerebral infection. The reason for the dissemination of TMEV within the SC associated with delayed virus elimination may be related to several factors including a different cell tropism with earlier involvement of microglia/macrophages thereby enabling and fostering an extended virus persistence and spread. The regulation of virus persistence includes a complex virus-host interaction, which is determined among others by the effectiveness of the antiviral innate and adaptive immune response in combination with the genetic background of the mouse strain^46–48^.

Resistant B6 mice without experimental manipulation e.g. CD8 ablation^49^ neither develop demyelinating lesions within the brain nor in the SC following intracerebral infection due to a rapid virus elimination triggered by a strong antiviral immune response^14,40,43^. However, the present study shows that the localization of infection (brain versus SC) has a major impact upon the pathogenesis of TME since intraspinal infection of B6 mice was followed by demyelinating SC lesions as well as inflammatory and degenerative lesions within the PN. The observed differences in the pathogenesis of TME following i.s. versus i.c. infection may be related to region specific differences of the immune response as also described in other studies leading to a prolonged presence of TMEV within the CNS^16,18,20,22,50^. After i.s. infection of B6 mice, a caudal and rostral dissemination of inflammation was detected with demyelinating lesions in association with inflammation. Significant demyelination occurred at 14 dpi, coinciding with the most prominent inflammatory changes.

Virus persistence in the SC of susceptible mouse strains is an important requirement for the development of demyelinating SC lesions since the underlying mechanisms are suggested to be immune-mediated including epitope spreading as well as molecular mimicry^15,51^. Previous studies in B6 mice showed that virus persistence could be prolonged and clinical sings induced by combining intracerebral infection with intraperitoneal administration of bacterial LPS or interleukin-1^52^. Therefore, in the present study virus persistence may have contributed to spinal cord lesions by similar mechanisms as observed in susceptible mice.

In the CNS, demyelinating events are followed by an initial phase of OPC recruitment followed by differentiation of NG2-positive OPCs into myelinating oligodendrocytes^53,54^. Demyelinating lesions in the chronic phase of TME in susceptible SJL mice show increased numbers of NG2-positive cells but - at the same time - only a very limited amount of remyelination mainly by oligodendrocytes and - to a lesser extent - Schwann cells^6,55–57^. In the present study, a lesion-associated, significantly increased number of tdTomato-positive cells in TMEV-infected mice was detected in the thoracic spinal cord at 28 compared to 14 dpi. This finding is consistent with other studies investigating different forms of SC injury and may be interpreted as an early regenerative approach^58,59^. In addition to perilesional tdTomato-positive cells, a sparse, accumulation of PRX-positive Schwann cells was detected indicating a contribution of Schwann cells to remyelination in B6 mice as known from SJL mice during chronic TME^60^.

### Peripheral nerves

Intracerebral TMEV infection of resistant B6 mice leads to significant lesions in the CNS, mainly an acute polioencephalitis, while lesions in peripheral organs including PN were not detected^40^. The degree of SC inflammation and demyelination decreased after 14 dpi in the present study while the clinical impairment remained on a high level. These findings in combination with the emergence of a peripheral neuropathy were rather surprising. PN showed degenerative as well as inflammatory lesions characterized by a dominant infiltration of macrophages. In addition, axonal degeneration indicated by an accumulation of β-APP as well as a significant demyelination was observed within the PN at 14 and 28 dpi. In the present study, PN lesions may be triggered by both, an outside-in as well as an inside-out mechanism^61^. Virus antigen was detected in single nerve fibers of 2/26 TMEV infected animals (one at 7 and one at 14 dpi) indicating that virus spread from the SC to the PN represents a rare event. This raises the question whether the pathomechanism of PN damage is related to a spread of inflammation from the CNS to the PNS or the inflammatory response within the PN is part of a compartmentalized, CNS independent peripheral immune response.

Diseases of the PNS are classified with regard to the affected components, speed of development, causative agents and clinical signs. GBS is a rare but potentially life threatening disease with several subtypes characterised by primary or secondary axonal degeneration and myelin loss within the PNS resulting in neurological impairment like flaccid paralysis and areflexia^32–34^. The different subtypes are divided into an acute inflammatory demyelinating neuropathy (AIDP), representing the most common type of GBS, and acute motor/motor-sensory axonal forms (AMAN/AMSAN) with regard to the types of nerve fibers involved and the predominant manifestation of fiber injury demyelinating versus axonal^62,63^. Within AIDP the proximal parts of the PN are affected by lymphocytic inflammation and macrophage-associated demyelination whereas AMAN/AMSAN show only little numbers of lymphocytes as well as sparing of dorsal nerve roots and dorsal root ganglia^63^. Campylobacter jejuni, the leading cause of acute gastroenteritis in developed countries is the most frequently suspected causative agent but several other bacterial as well as viral pathogens are thought to be possible antecedent triggers of GBS^34^. Recent studies suggest a causal link between ZIKA virus infection as a predisposition for GBS pointing out a concomitant high actuality of virus-triggered neuropathies^64,65^. Marek’s disease (MD), a herpesvirus infection in chicken, is known to entail a demyelinating disease of PN resembling those seen in experimental autoimmune neuritis (EAN), the first known animal model for GBS^66^. Despite the high number of potential viral pathogens, only few virus-triggered models of GBS are available and have been described in detail. The observed peripheral nerve lesions in intraspinally TMEV infected mice share several features of GBS such as axonal damage, inflammation and demyelination thereby representing a valuable tool to study virus-mediated PN lesions in immunocompetent B6 mice.

## Conclusions

In this study, B6 mice developed significant clinical signs, demyelinating lesions in the SC as well as PN lesions without any additional treatment like immunosuppression or immune stimulation. Major advantages of i.s. infection are the short time span between infection and lesion development in the SC as well as the emergence of a peripheral neuropathy. PN lesions including axonal damage, macrophage infiltration and demyelination shared features of GBS resembling the primary axonal types (AMAN/AMSAN) with a macrophage dominated, inflammatory response. Therefore, this model may serve as a suitable tool for further investigations of virus triggered PN lesions.

## Material and Methods

Four to five week old female C57BL/6.NG2CreERT2xRosa26.floxed.stop-tdTomato double heterozygous mice^27^ were randomly assigned to groups using the random function in Microsoft Excel. Animals were continuously fed with a special diet containing tamoxifen citrate (TD55125, 400 mg/kg tamoxifen citrate; envigo, Indianapolis, United States) ad libitum, starting at seven days prior to i.s. TMEV inoculation, inducing a permanent tdTomato expression in NG2-Cre-transgenic cells. Animals (n = 51) were randomly assigned to treatment groups and housed in an individually ventilated cage system (Tecniplast, Hohenpeißenberg, Germany) with free access to drinking water. Group size varied from five to eight animals for each investigated time point and treatment (infected vs. mock).

### Infection and virus

A hemilaminectomy and stereotaxic inoculation at the level of the tenth thoracic vertebra was performed as previously described^67^. Animals were i.s. infected with 4.56 × 10^3^ plaque-forming units of the BeAn strain of TMEV/animal. The control group (mock) received an equivalent fluid volume of cell culture supernatant. Surgical procedures were performed under general anaesthesia using medetomidine (0.05 mg/kg; Domitor®, Orion Pharma, Espoo, Finland) and ketamine (10 mg/kg; Ketamin 10%, bela-pharm, Vechta, Germany) as well as analgetic treatment with Tramadol-Ratiopharm® (15 mg/kg; Ratiopharm, Ulm, Germany) and Carprofen (4 mg/kg; Rimadyl®, Pfizer, New York City, New York, United States). During anaesthesia, eyes were covered with ointment (Bepanthen® Augen- und Nasensalbe, Bayer AG, Leverkusen, Germany). After operation, animals received analgesia via drinking water (Tramadol-Ratiopharm®, 1 mg/ml; Ratiopharm, Ulm, Germany).

### Clinical investigation

Clinical investigation was performed daily and covered the categories posture and external appearance (0 = normal posture; smooth and shiny coat, firmly lying to the body; 1 = normal posture; shaggy and dull coat; 2 = mildly hunched back; shaggy and dull coat; 3 = markedly hunched back; shaggy, dull and soiled coat), behaviour and activity (0 = attentive and curious; 1 = very calm: mildly reduced spontaneous locomotion, no reduced induced movement; 2 = apathy: moderately reduced spontaneous locomotion, mildly reduced induced movement; 3 = stupor: no spontaneous locomotion, little induced movement) and gait (0 = normal motion sequence; 1 = mild paresis of hind limbs: occasionally observable mild unsteady gait (swaying, stumbling, falling, shortened steps); partially only apparent when climbing at cage; 2 = moderate to severe paresis of hind limbs: frequently observable unsteady gait (swaying, stumbling, falling, shortened steps); 3 = severe paresis of hind limbs or spinal ataxia: frequently observable moderate to marked unsteady gait (swaying, stumbling, falling, shortened steps); delayed standing up from supine position; 4 = paralysis of one hind limb or high-grade spinal ataxia; spastic paresis of more than one limb; marked delay in standing up from supine position). The clinical score represents the sum of the three categories.

### Rotarod performance test

For rotarod performance test (RotaRod Treadmill, TSE Technical & Scientific Equipment, Bad Homburg, Germany), mice were trained at −5 dpi and −3 dpi for 10 minutes each with a constant speed of 5 or 10 rounds per minute (RPM), respectively. Investigations at 0 (before surgery), 7, 14 and 28 dpi were performed using an accelerated rotarod test (increased rotation speed from 5 to 55 RPM over a time span of 5 minutes). For statistics, a mean value of three consecutive runs was calculated.

### Tissue processing

Animals were perfused with phosphate buffered saline (PBS). Tissue samples of SC including injection site (T9–11), cervical (C1–C3) and lumbar (L3-S4) segments, PN (proximal and middle part of plexus brachiales and nervi ischiadici) as well as other organs (esophagus, trachea, thyroid, parathyroid, thymus, heart, spleen, liver, stomach, pancreas, small and large intestine, lymph nodes (mesenteric, subiliac, cervical, mandibular, lumbar, mediastinal), kidneys, genital organs including mammary gland, urinary bladder, skeletal muscle, skin, salivary gland, tongue, nose, bone and bone marrow) were formalin fixed and paraffin embedded (FFPE) for further investigation. Serial slices of SC and PN were cut and subsequently stained using hematoxylin and eosin (HE). In addition, immunohistochemistry was performed using a marker panel summarized in Table 1.

**Table 1:** Antibodies used for immunohistochemistry.

Antigen, Target	Company; product number; clone	Blocking Serum	Pre-treatment	Dilution	2^nd^ antibody (1:200)
TMEV	—	Goat	—	1:2000	Goat anti-rabbit
LivingColors DsRed, TdTomato	Takara Bio Europe/Clontech 632496	Goat	Microwave, 20 minutes, citrate buffer	1:500	Goat anti-rabbit
β-APP, axonal damage	Merck/Millipore MAB348	Goat	Microwave, 20 minutes, citrate buffer	1:2000	Goat anti-mouse
Periaxin, Schwann cells	Sigma HPA001868	Goat	Microwave, 20 minutes, citrate buffer	1:5000	Goat anti-rabbit
CD3, T lymphocytes	Dako/Agilent Technologies A0452	Goat	Microwave, 20 minutes, citrate buffer	1:250	Goat anti-rabbit
CD45R, B lymphocytes	BD Bioscience 553085	Goat	Microwave, 20 minutes, citrate buffer	1:1000	—
CD107b, microglia/macrophages	Bio Rad MCA2293	Goat	Microwave, 20 minutes, citrate buffer	1:400	Goat anti-rat
MBP, myelin	Chemicon AB980	Goat	—	1:500	Goat anti-rabbit

TMEV = Theiler’s murine encephalomyelitis virus; β-APP = beta-amyloid-precursor protein; MBP = myelin basic protein.

### Immunohistochemistry

Immunohistochemistry was performed as previously described^6,68,69^. Antigen retrieval, if required, was performed by incubating slides in boiling citrate buffer in a microwave, followed by application of inactivated goat serum. Avidin-biotin-peroxidase reagent (Vectastain ABC Kit; Vector Laboratories, Burlingame, California, United States) as well as 3,3′-Diaminobenzidine tetrahydrochloride (Sigma-Aldrich, St. Louis, Missouri, United States) were used for visualization of antigen-antibody reactions. For visualization of TMEV (virus protein), tdTomato-positive cells (OPCs and matured stages), beta-amyloid precursor protein (β-APP; axonal damage), Periaxin (PRX, Schwann cells), CD3 (T lymphocytes), CD45R (B lymphocytes), CD107b (microglia/macrophages) and myelin basic protein (MBP; myelin) primary antibodies (Table 1) were diluted in PBS containing 1% bovine serum albumin.

### Histological examination

Inflammation was semiquantitatively evaluated on HE stained transversal SC sections. The applied scoring system included four identical categories for white and grey matter (0 = normal; 1 = single perivascular infiltrates; 2 = 2–3 layers of perivascular infiltrates; 3 = >3 layers of perivascular infiltrates). Semiquantitative scores were averaged for each category and cross section.

For immunohistochemical detection of TMEV, CD3, CD45R, CD107b, Periaxin and β-APP within the SC, positive cells or axons were counted manually for each cross section, respectively. TdTomato-positive glial cells were also manually counted and quantified in the thoracic segment. Quantification of demyelination was performed using analySIS® 3.2 software (SOFT Imaging System; Olympus, Münster, Germany). Slides were digitalized, MBP-negative areas manually outlined and quantified.

PN were investigated for inflammatory and degenerative changes using HE staining and immunohistochemistry. For CD3, CD45R, CD107b as well as β-APP the density of positive cells or axons per mm² was calculated. Morphometry quantifying myelin loss was performed using analySIS® in the most affected HPF.

### Statistics

Statistical analysis was done using SPSS for Windows version 24 (IBM® SPSS® Statistics, SPSS Inc., Chicago, IL, United States). Normal distribution was tested via Kolmogorov-Smirnov- and Shapiro-Wilk-test, followed by Mann-Whitney-U post hoc tests. Statistical significance was accepted at a p-value of  ≤0.05.

### Ethics statement

Animal experiments were conducted in accordance with the German Animal Welfare Law and all experiments were approved by the local authorities (Niedersächsisches Landesamt für Verbraucherschutz und Lebensmittelsicherheit (LAVES), Oldenburg, Germany, permission number: 33.12-42502-04-15/1996).
